# Supplementary material for: The genetic relationships between brain structure and schizophrenia
Source: Nat Commun. 2023 Nov 28;14:7820. doi: 10.1038/s41467-023-43567-7 (PMC10684873; doi:10.1038/s41467-023-43567-7)
Supplement: Supplementary file 1 — Supplementary Information [file 41467_2023_43567_MOESM1_ESM.pdf]

Supplementary Notes: The genetic relationships between brain structure and schizophrenia

Eva-Maria Stauffer[1,*], Richard A.I. Bethlehem[1,2], Lena Dorfschmidt[1], Hyejung Won[2], Varun Warrier[1,†], and Edward T. Bullmore[1,4†]

[1]Department of Psychiatry, University of Cambridge, Cambridge, UK
[2]Department of Psychology, University of Cambridge, Cambridge, UK
[3]Department of Genetics and the Neuroscience Center, University of North Carolina at Chapel Hill, Chapel Hill, NC, USA
[4]Cambridgeshire & Peterborough Foundation NHS Trust, Cambridge, UK
*corresponding author(s): Eva-Maria Stauffer (ems206@cam.ac.uk)
†these authors contributed equally to this work

Contents

1 Magnetic resonance imaging (MRI) data acquisition, preprocessing, and analyzable sample 3

2 Genetic intersection of brain structure and schizophrenia was robust to gene inclusion criteria 4

3 Normative structural covariance SC and genetic similarity GS networks 5

3.1 Effects of physical distance on SC and GS . . . 5
3.2 Mapping of SC and GS networks to Mesulam atlas of cortical cytoarchitectonics . . . 5
3.3 Mapping of SC and GS networks to Yeo atlas of functional networks . . . 6
3.4 Effects of measurement reliability on regional degree . . . 6

4 Characterisation of spatial PLS1 maps 9

4.1 Pleiotropic associations with MRI phenotypes were strongest for regional nodes with highest intra-modular or inter-modular degree . . . 9
4.2 Enrichment of PLS1 weights in known cortical atlases . . . 9

5 Gene enrichment analyses 11

5.1 Thresholds for $\Delta(R(T,U))$ used to define gene sets for enrichment analysis . . . 11
5.2 Enrichment for constrained genes and cell types . . . 11

6 Specificity of schizophrenia's intersection with MRI-associated genes 13

7 Mendelian randomization 14

List of Figures

S1 **Distance and structural covariance.** . . . 5
S2 **Structural covariance and genetic similarity.** . . . 5
S3 **Relationship of SC and GS to Mesulam classes.** . . . 6
S4 **Relationship to Yeo networks.** . . . 7
S5 **Measurement reliability and regional degree.** . . . 8
S6 **Brain regions pleiotropically associated with schizophrenia are hubs of normative structural covariance networks.** . . . 9
S7 **PLS1 weight enrichment.** . . . 10
S8 **Thresholds for pleiotropic gene sets for enrichment analyses.** . . . 11
S9 **Enrichment for constrained genes and cell types.** . . . 12
S10 **Phenotypic relationships between height and MRI metrics** . . . 13

S11 **Mendelian randomization plots for surface area of cortical areas, ProS and V4.** 15

1 Magnetic resonance imaging (MRI) data acquisition, preprocessing, and analyzable sample

To generate structural covariance matrices we accessed MRI data from the UK Biobank [1]. We focused on a subset of N = 40,680 participants for each of whom complete genotype and multimodal MRI data were available for download (February 2020). MRI data acquisition has been described in detail elsewhere [2]. In brief, MRI data was collected on a 3T Siemens Skyra scanner (Siemens, Munich, Germany) using a 32-channel receive head coil. T1-weighted images were acquired using a 3D MPRAGE sequence with the following key parameters; voxel size 1x1x1mm, TI/TR = 880/2000 ms, Field-of-view = 208x256x256 matrix, scanning duration: five minutes. The diffusion imaging data was acquired using a monopolar Steejskal-Tanner pulse sequence and multi-shell acquisition (b=0s/mm2, b=1,000s/mm2, b=2,000s/mm2) with the following key parameters; voxel size 2x2x2mm, TE/TR = 92/3600 ms, Field-of-view = 104x104x72 matrix and scanning duration = seven minutes [2].

We followed preprocessing steps outlined in [3]. In brief, minimally processed T1- and T2-FLAIR- weighted MRI data (and DWI data) were downloaded from UK Biobank (application 20904) and further processed with Freesurfer (v6.0.1) [4] using the T2-FLAIR weighted images to improve pial surface reconstruction. Preprocessing steps included bias field correction, registration to stereotaxic space, intensity normalization, skull-stripping, and grey/white matter segmentation; Following reconstruction, the Human Connectome Project (HCP) parcellation [5] was aligned to each individual image and regional metrics were estimated for 180 bilateral cortical areas. Neurite orientation dispersion and density imaging (NODDI) reconstruction was performed using the AMICO pipeline [6].

We excluded participants with incomplete MRI data and we additionally excluded participants who were robustly defined as outliers by global or regional metrics more than 5 times the median absolute deviation from the sample median ($\pm$ 5 MAD). For CT and SA this lead to 31,780 subjects and 31,797 subjects for NDI.

In the present analyses we focused on MRI metrics that have been widely used and well-validated in the neuroimaging community, with multiple studies by the UK Biobank consortium providing the data [7, 8, 9], including results which have demonstrated that all three metrics have high levels of test-retest reliability in a repeatedly scanned subset of the UKB MRI cohort [10].

2 Genetic intersection of brain structure and schizophrenia was robust to gene inclusion criteria

Genes can be identified using multiple different approaches with varying levels of inclusivity. For example, Trubetskoy et al. published a broad fine-map set of schizophrenia genes which included 628 (435 protein-coding) genes as well as a prioritized list of 120 genes (106 protein-coding) [11]. To ensure that the identified genes which overlapped between schizophrenia and brain structure were robust to the method used for gene identification, we performed three sensitivity analyses. First, we investigated the overlap between the 587 schizophrenia-associated genes we identified and the two lists of schizophrenia-associated genes reported by Trubetskoy et al., (106 protein-coding genes and a more inclusive list of 435 genes) [11]. Second, we investigated the gene-level overlap between the 106 schizophrenia-associated genes with the list of brain MRI-associated genes published by Warrier et al.[3]. Third, because the number of genes significantly associated with MRI phenotypes was low, due to the relatively small sample size of MRI GWAS, we additionally performed enrichment analysis to test whether MRI-associated genes were enriched for the 106 schizophrenia-associated genes.

First, we found that out of the 106 protein-coding genes reported by Trubetskoy et al. [11], 42 genes (40%) were also included in our list of genes associated with schizophrenia. Additionally, out of the 435 protein-coding genes listed in the more inclusive set of schizophrenia-associated genes [11], 198 (46%) were also identified by our analysis. Importantly, some of the genes robustly associated with schizophrenia across these various lists were genes that showed strong effects on the genetic covariation between schizophrenia and all MRI phenotypes, e.g., *CRHR1*, *KANSL1* and *MAPT* on chromosome 17q21 and *ATG13* on chromosome 11p11. Second, the prioritised gene lists for MRI phenotypes reported by Warrier et al. [3] included 16 genes for SA, 12 for CT and two for NDI, all of which were also identified using our gene-level analysis. From these two publications [3, 11], we identified four genes that were robustly associated with schizophrenia and with SA, three genes with CT, and three with NDI. These consistently overlapping genes included *BNIP3L* on chromosome 8p21 and *CRHR1*, *MAPT* and *KANSL1* on chromosome 17q21. Third, we found that gene effects for surface area and cortical thickness were enriched for 106 fine-mapped genes for schizophrenia (surface area $Z = 2.6$, $P < 0.02$; cortical thickness $Z = 2.6$, $P < 0.01$). Gene-level effects for neurite density index were not significantly enriched for schizophrenia-related genes ($Z = 0.8$, $P = 0.4$). Taken together, these additional analyses support our findings of overlap between schizophrenia- and MRI-associated genes. Specifically, using more stringent gene lists for both schizophrenia and MRI phenotypes, we replicated the overlap of schizophrenia- and brain-related genes on chromosome 17q21.

3 Normative structural covariance SC and genetic similarity GS networks

3.1 Effects of physical distance on SC and GS

Similar to genetic similarity, structural covariance was negatively associated with geodesic distance. Shown are distance decay functions for structural covariance matrices for surface area, cortical thickness and neurite density index (Fig. S1). We estimated Spearman's correlations between structural covariance and genetic similarity after accounting for linear effects of distance between each pair of structurally covarying or genetically similar regions nodes. As shown in Figure S2, the correlations between structural covariance and genetic similarity remained high, indicating that the strong coupling between phenotypic covariance and genetic correlation is not largely driven by the potentially confounding effect of physical distance between nodes.

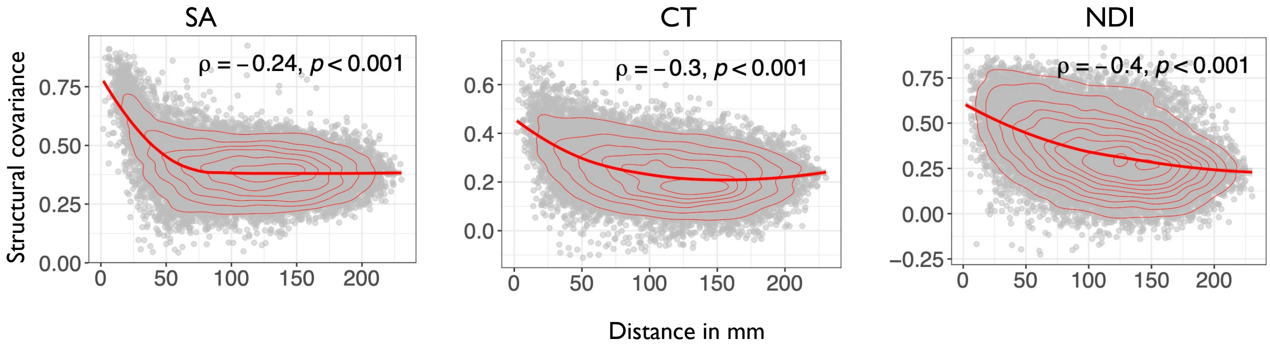

Figure S1. Distance and structural covariance. Distance decay function for surface area, cortical thickness and neurite density index based on structural covariance. Two-tailed correlation (Spearman's) between structural covariance (y-axis) and geodesic distance in millimeters (x-axis).

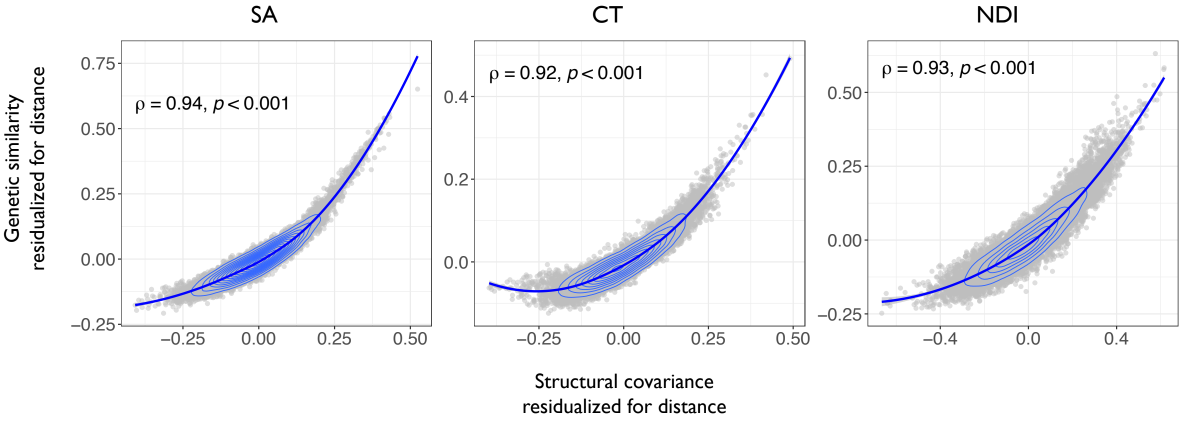

Figure S2. Distance effects on the relationship between structural covariance and genetic similarity. Edge-wise two-tailed Spearman's correlation between genetic similarity (y-axis) and structural covariance (x-axis) matrices adjusted for distance effects.

3.2 Mapping of *SC* and *GS* networks to Mesulam atlas of cortical cytoarchitectonics

Structural covariance and genetic similarity showed similar relationships to classes of laminar differentiation [12], echoing the finding that genetic similarity and structural covariance are highly similar. For CT structural covariance and genetic similarity were enriched in idiotypic and heteromodal class (Fig. S3). Genetic similarity of cortical thickness was higher in heteromodal ($z = 3.94$, $P < 0.05$) and idiotypic ($z = 2.85$, $P < 0.05$) classes. Structural covariance showed the same relationship cytoarchitectonic classes (CT heteromodal $z = 3.46$, $P < 0.05$; idiotypic $z = 2.48$, $P < 0.05$).

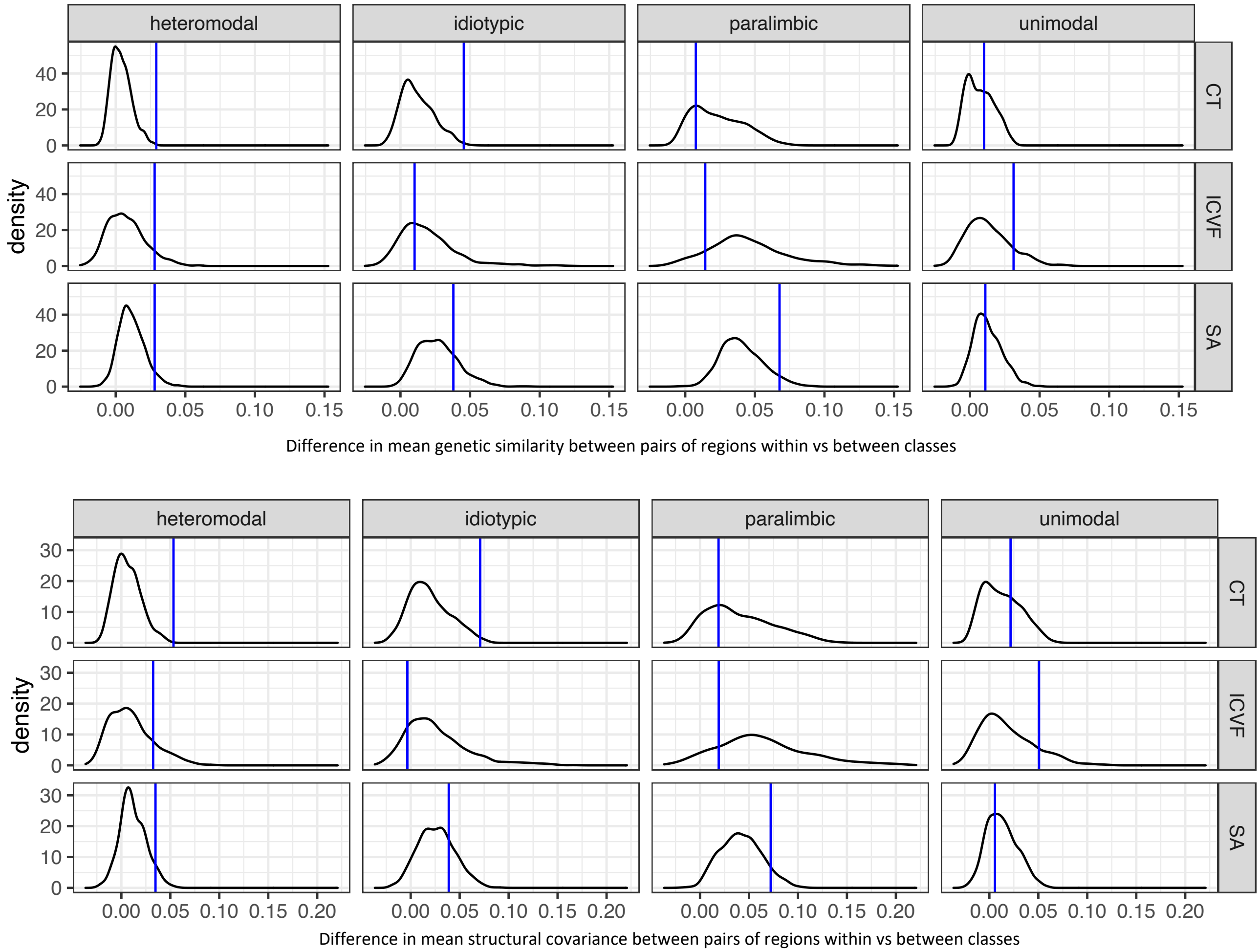

Figure S3. Relationship of structural covariance and genetic similarity to Mesulam's cytoarchitectonic classes. We tested whether genetic similarity or structural covariance were higher or lower than expected by chance between regions of the same cytoarchitectonic class compared to regions in different classes[13]. Significance was assessed by two-tailed spin-permutation. Blue lines indicate true value of the difference in mean genetic similarity or structural covariance between pairs of regions within versus between classes.

3.3 Mapping of *SC* and *GS* networks to Yeo atlas of functional networks

Structural covariance and genetic similarity showed similar relationships to Yeo networks[14]. For SA structural covariance and genetic similarity were enriched in sensory-motor and default mode networks. Additionally genetic similarity for SA was enriched in dorsal attention networks and CT in fronto-parietal networks (Fig. S4). For surface area, genetic similarity was higher among regions within the sensory-motor (z = 3.24, $P < 0.05$) and default mode (z = 2.84, $P < 0.05$) networks relative to random networks. Structural covariance showed the same relationship to networks (SA sensory-motor z = 3.03, $P < 0.05$; default mode z = 2.6, $P < 0.05$).

3.4 Effects of measurement reliability on regional degree

To ensure that regional degree (hubness) was not merely a reflection of measurement reliability, we investigated the relationship between measurement reliability and regional degree in a subset of the UK Biobank imaging sample. To this end, we assessed a subset of subjects from the UK Biobank for whom repeat scans were available (N = 1,363). Only T1 follow-up scans were only available for this analysis, thus we estimated CT and SA. We performed the same imaging quality controls as for the baseline scans. We excluded subjects with incomplete imaging data and subjects that were identified as global or regional outliers, i.e. more than five times the median absolute deviation from the sample median. For CT and SA, this led to N = 1,280 subjects with both baseline and follow-up scans. To assess test-retest reliability of the regional MRI metrics, we estimated the two-tailed (Pearson's) correlation between the group average baseline and follow-up measurements of SA or CT for each brain

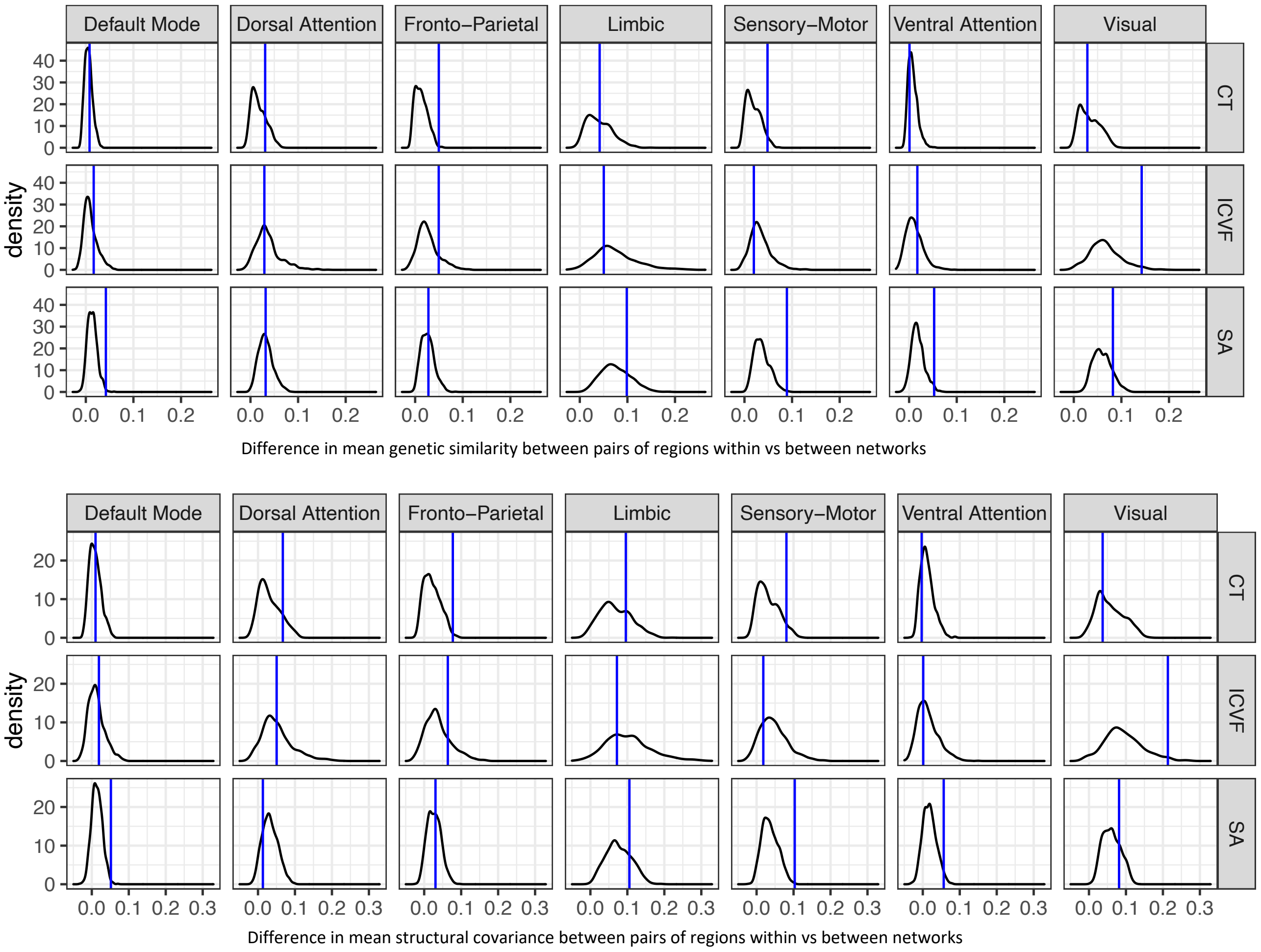

Figure S4. Enrichments of structural covariance and genetic similarity in Yeo networks. We tested whether genetic similarity or structural covariance were higher or lower than expected by chance within regions of the same Yeo network [14] compared to regions between Yeo networks. Significance was assessed by spin-permutation. Shown are permutation results from enrichments tests for Yeo networks. Blue lines indicate true value of the difference in mean genetic similarity or structural covariance between pairs of regions within versus between networks.

region. For CT, the test-retest reliability ranged between $0.5 \geq R \leq 0.9$ (mean $R = 0.78$) and for SA $0.71 \geq R \leq 0.99$ (mean $R = 0.97$). Thus, in line with previous findings, the test-retest reliability of these MRI metrics is good to excellent [10]. Finally, we calculated Pearson's correlations between test-retest reliability and regional degree based on structural covariance networks derived from the baseline CT or SA data. As shown in Figure S5, there was no significant correlation between measurement reliability and regional degree (CT, $R = 0.1$, $P = 0.17$; SA, $R = -0.06$, $P = 0.4$). These results suggest that regional degree is not driven by test-retest reliability of MRI metrics.

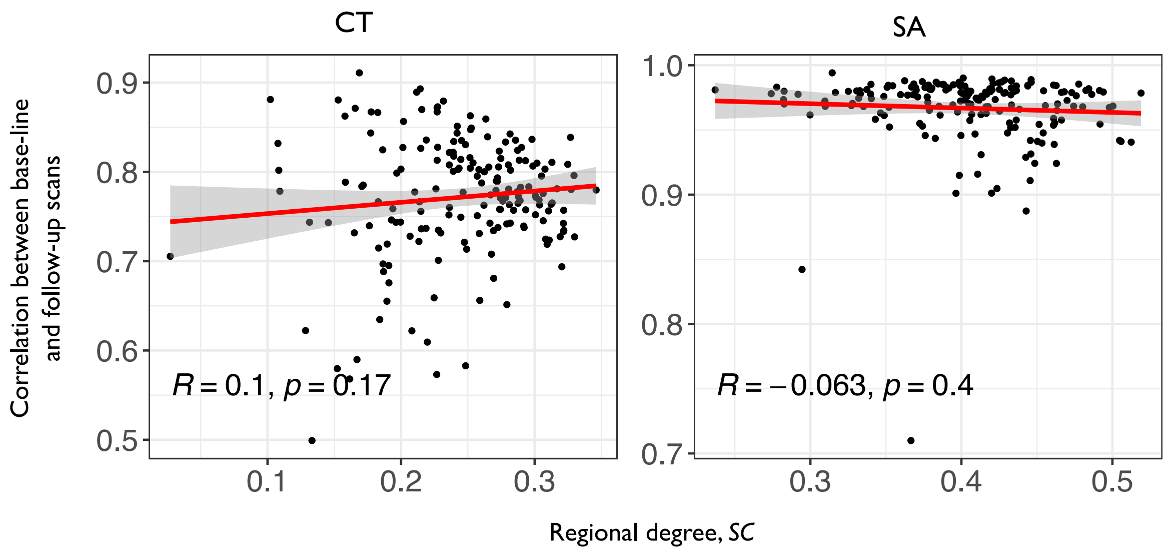

Figure S5. Measurement reliability and regional degree. Scatterplots of measurement reliability, indexed by two-tailed Pearson's correlations between baseline and follow-up scans (y-axis), and regional degree based on structural covariance networks (x-axis) for cortical thickness (CT) and surface area (SA). Each data point represents one of 180 brain regions.

4 Characterisation of spatial PLS1 maps

4.1 Pleiotropic associations with MRI phenotypes were strongest for regional nodes with highest intra-modular or inter-modular degree

PLS1 weights showed significant correlations with intra-modular and inter-modular degree based on normative networks (Fig. S6).

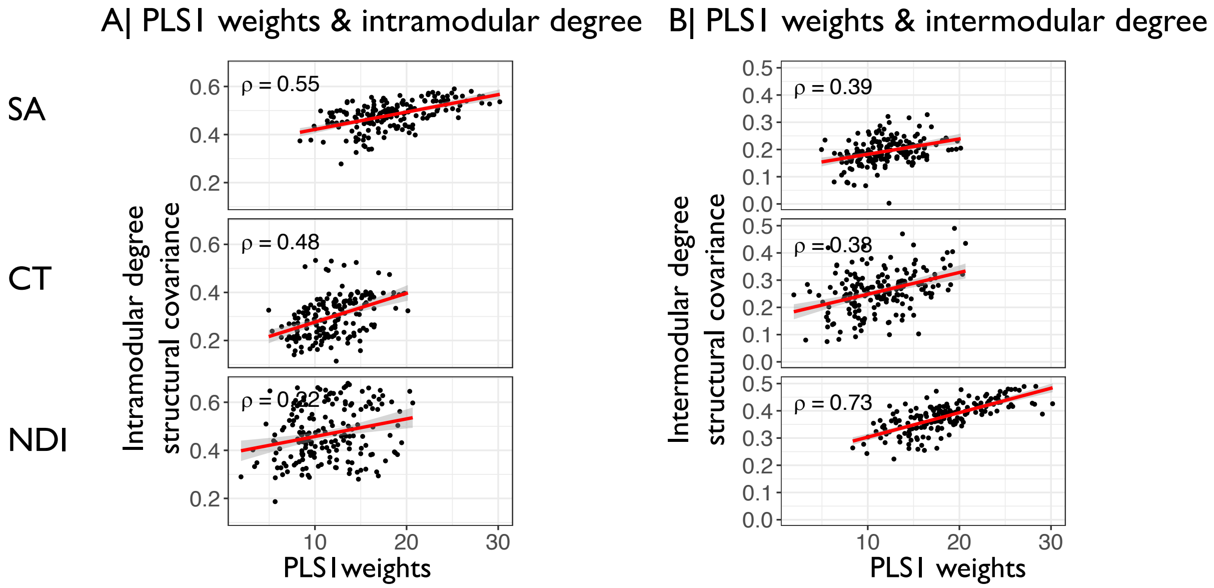

Figure S6. Brain regions pleiotropically associated with schizophrenia are hubs of normative structural covariance networks. Scatterplots of PLS1 weights (x-axis) versus intra-modular degree (A, ya-xis) or inter-modular degree (B, y-axis) for surface area (SA), neurite density index (NDI) and cortical thickness (CT). Inter- and intra-modular degree were based on modular decomposition of structural covariance matrices by the Louvain alrorithm (see Fig 4). We found significant positive Spearman's correlations for all MRI metrics, i.e., higher degree or "hubness" was correlated with increasing surface area, thickness or neurite density of cortical macro- or micro-structure.

4.2 Enrichment of PLS1 weights in known cortical atlases

As outlined in the main text we investigated whether PLS1 weights were related to two predefined and one data driven cortical atlases. Specifically, we tested for enrichment in Mesulam classes [12], Yeo networks [14] and modules based on structural covariance and genetic similarity networks. Distributions were generated by spin-permutation. PLS1 weights were higher than expected by chance in the paralimbic class for SA ($Z = 2.77$, $P < 0.05$); in the heteromodal class ($Z = 2.15$, $P < 0.05$) and idiotypic class of cortex ($Z = 2.61$, $P < 0.05$) for CT; and in the heteromodal class for NDI ($Z = 2.69$, $P < 0.05$). We did not find any significant enrichments of PLS1 weights for functional networks. However, PLS1 weights were significantly enriched for CT in module two (Fig. S7).

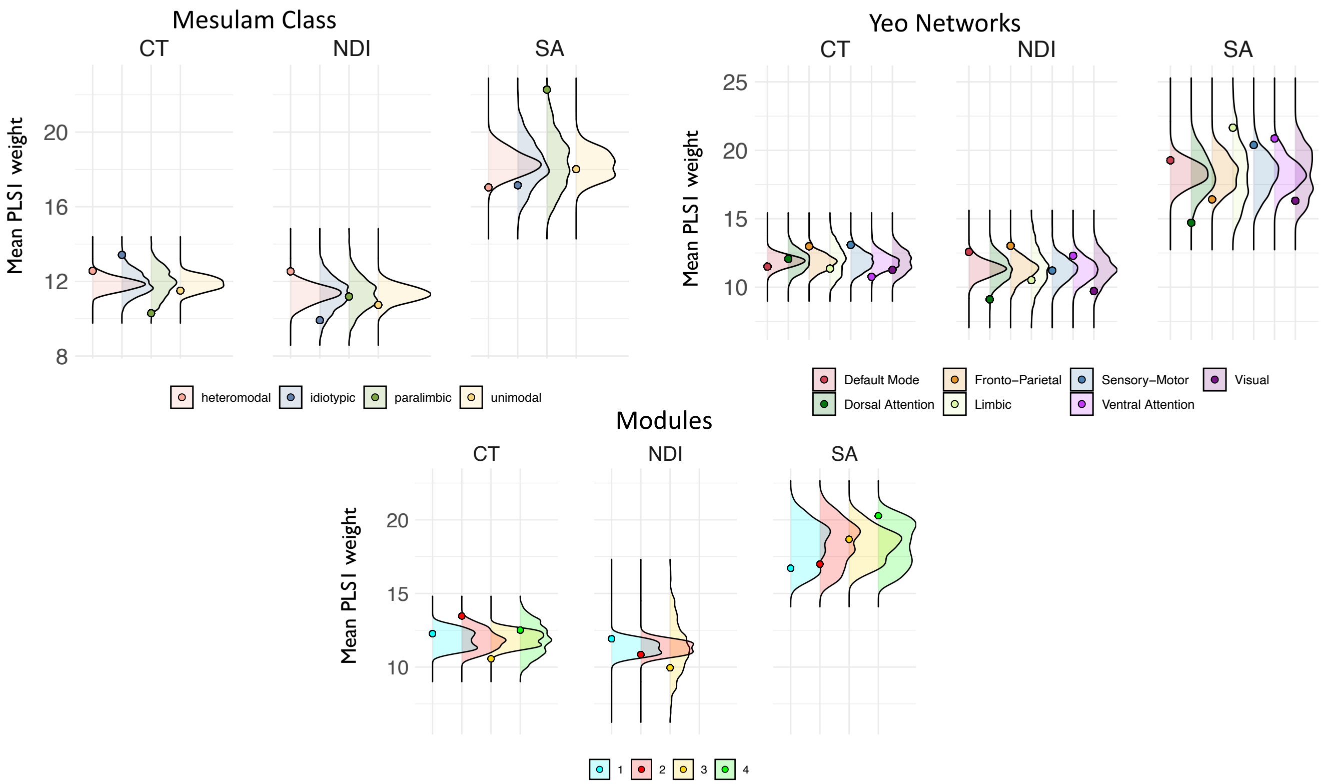

Figure S7. **PLS1 weight enrichment.** Enrichment of PLS1 weights for cortical thickness, neurite density index and surface area in Mesulam classes [12], Yeo networks [14] and genetically informed modules. Distributions of mean PLS1 weights were generated using spin-permutations. Circles indicate the true value.

5 Gene enrichment analyses

We used multiple different enrichment tests in several different gene sets of interest. The principal gene set of interest was the set of the top 1% (or 3%) most pleiotropic genes, with pleiotropic association of each protein-coding gene defined by it's $\Delta(R(T,U))$ score in leave-one-out PLS analysis. Other gene sets of interest included genes significantly and independently associated with clinical (schizophrenia, bipolar disorder, Alzheimer's disease) or brain structural (regional SA, CT, NDI) phenotypes.

5.1 Thresholds for $\Delta(R(T,U))$ used to define gene sets for enrichment analysis

Since both schizophrenia and brain structural phenotypes are polygenic and likely influenced by genes that do not reach genome-wide significance, we considered it important to go beyond the characterisation of a small number of genes and additionally to characterise gene sets with large pleiotropic effects. However, as shown in Figure S8A, many of the genes showed weak effects which would bias downstream enrichment analysis. We therefore selected genes based on thresholds for $\Delta(R(T,U))$ chosen to avoid the inclusion of genes that have no or very low pleiotropic effects on brain MRI phenotypes and schizophrenia (or any other clinical disorder for which there is GWAS data). We thus included a larger proportion of genes with a relatively strong effect on the covariation between schizophrenia and brain structure (top 1%) to perform gene set enrichment analysis. This first threshold is conservative and only includes 185 genes with pleiotropic effects indexed by $\Delta(R(T,U)) \geq 2$ or $\leq$ -2 (Fig.S8B). To ensure that the reported enrichment findings are robust across different inclusion thresholds, we widened the analysis to include the top 3% of genes. This threshold was chosen because it allows the inclusion of more genes (556) with effects indexed by $\Delta(R(T,U)) \geq 1$ or $\leq$ -1 (Fig.S8 C). As reported in the main paper, enrichment results based on the top 1% gene set were robustly replicated in the top 3% gene set.

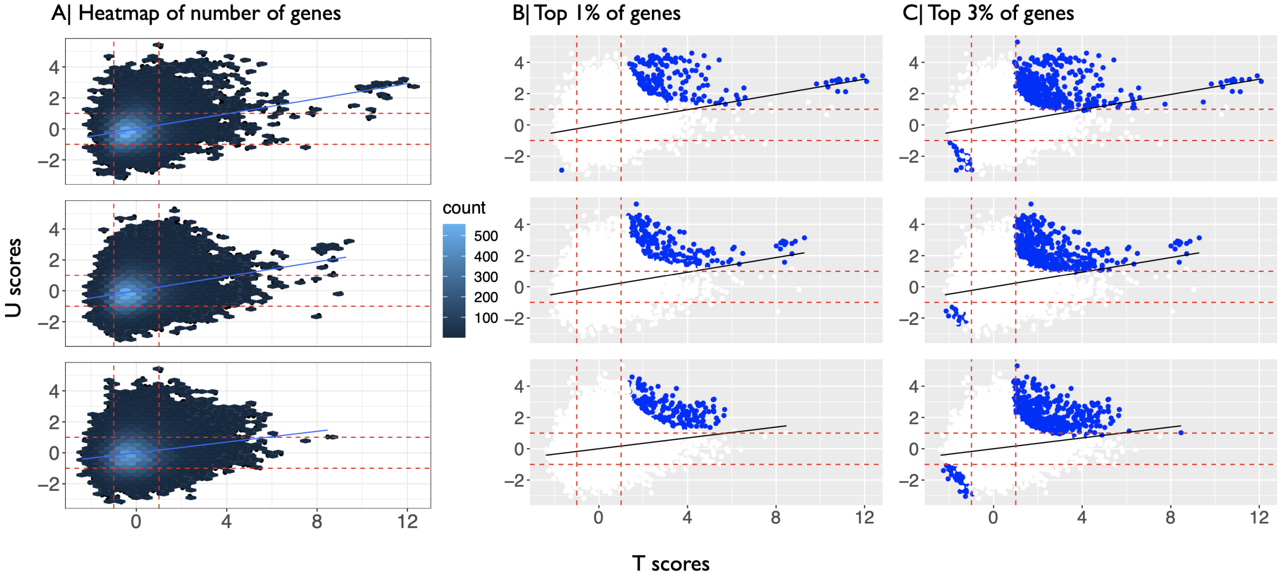

Figure S8. Thresholds for pleiotropic gene sets for enrichment analyses. (A) Scatterplot of T scores (x-axis) versus U scores (y-axis) for each of 18,640 protein-coding genes, derived from their weights on the first PLS component. The number of genes is shown as a heatmap (count). (B) Scatterplot of T scores (x-axis) versus U scores (y axis) for top 1% of genes with highest $\Delta(R(T,U))$ scores (see Methods). (C) Same as in (B) but for top 3% of genes on $\Delta(R(T,U))$. Red lines are drawn at effect sizes of 1 and -1.

5.2 Enrichment for constrained genes and cell types

We tested whether genes covarying between schizophrenia and MRI metrics were enriched for constrained genes or specific cell types. The analyses was performed on two inclusion thresholds and are shown in Figure S9A-B).

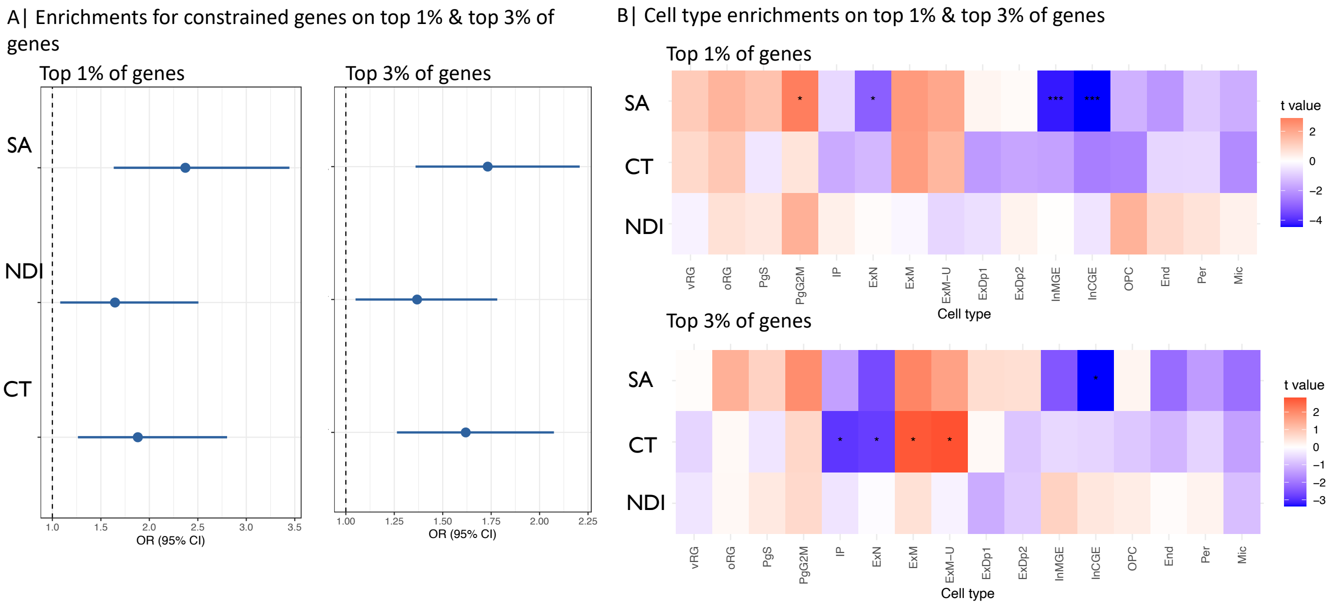

Figure S9. Enrichment for constrained genes and cell types. (A) Enrichment results for constrained genes for the top 1%and of genes with the highest influence on the covariation between brain structure and schizophrenia (highest for SA, CT and NDI. (B) Cell type enrichments of the top 1% and 3% of genes with the highest influence on the covariation between brain structure and schizophrenia. Cell types: vRG = ventral radial glia, oRG = outer radial glia, PgS and PgG2M = cycling progenitors, S phase and G2-M phase respectively, IP = intermediate progenitors, ExN = Migrating excitatory neurons, ExM = Maturing Excitatory neurons, ExMU = Maturing excitatory neuron, upper enriched, ExDp1 = Excitatory deep layer neurons 1, ExDp2 = Excitatory deep layer neurons 2, InMGE = MGE Interneuron, InCGE = CGE interneuron, OPC = oligodendrocyte precursor cells, End = endothelial cells, Per = pericytes.

6 Specificity of schizophrenia's intersection with MRI-associated genes

We compared the genes we identified as significant for bipolar disorder (BIP) and Alzheimer's disease (AD) to previously published fine-mapped or prioritized gene lists from prior GWAS to ensure that our lists overlap with fine-mapped genes. As outlined in the main manuscript, we found 136 genes were significantly associated with bipolar disorder after correction for multiple comparisons. 19 of them were also reported in the original paper [15] including prioritized genes such as *GNL3*, *TMEM258* and *STK4*. For Alzheimer's disease, we found 76 genes were significant after multiple comparisons correction. 55 of those genes overlapped with the 989 genes that were identified in the original publication [16] using position and expression quantitative trait loci, including high confidence genes such as *CD33* and *MADD*. For height, we identified 8,012 genes. The height GWAS was based on subjects of predominantly European ancestries and represents one of the largest, most well-powered GWAS's to date [17]. Out of 8,012 genes associated with height, 221 were also associated with SA, 96 genes with CT and 54 genes with NDI, representing a significant overlap. 21 genes were shared between height, SA, CT and NDI and were found on chromosome 17q21, 8p23 and chromosome 1p33.

As outlined in the main manuscript the first PLS component for each MRI metric identified a small but significant proportion of its genetically determined variation that covaried with genetic risks for bipolar disorder (2.7% for SA, 3.6% for CT and 1.6% for NDI). However, the variance explained for BIP was about 50% less than the variance explained for schizophrenia by the same MRI metrics; and the variance explained for AD was about 75% less than for schizophrenia (1.5% for SA, 1.2% for CT and 0.8% for NDI). The proportion of height-related variance was comparable to the proportion of schizophrenia-related variance across all MRI metrics (Height; SA = 7.1%, CT = 5.5%, NDI = 2.7%) (Fig.**??** A).

The strength of pleiotropic association with schizophrenia and MRI phenotypes, across all 18,640 genes, was also greater for schizophrenia than for BIP or AD. Specifically, the correlation between T and U scores decreased from schizophrenia, to bipolar disorder, to Alzheimer's disease. For height, we again found that the strength of pleiotropic association with SA was higher compared to schizophrenia, but similar to or lower than the strength of pleiotropic association with schizophrenia for CT and NDI (Fig. **??** C).

Finally, we visualised the intersection of the top 1% of genes with the highest $\Delta(R(T,U))$ values between schizophrenia and bipolar disorder, Alzheimer's disease, or height. The proportion of overlapping genes was less than 50% for bipolar and schizophrenia and further decreased for Alzheimer's disease and schizophrenia or for height and schizophrenia (Fig. **??** D). In summary, these results suggest that the genetic covariation between brain structure and schizophrenia is stronger than the genetic covariation between brain structure and bipolar disorder or Alzheimer's disease for all MRI metrics; and stronger than the genetic covariation between brain structure and height for most MRI metrics. More than 50% of the genes investigated in down-stream analysis (e.g. enrichment for constrained genes) were specific to schizophrenia. In this context we note that the top 1% of genes with the highest $\Delta(R(T,U))$ for height and CT, SA and NDI were not enriched for constrained genes ($p > 0.05$) compared to schizophrenia (Fig. S9).

Based on the results on the genetic relationship between height and SA, CT or NDI, we were stimulated to investigate the phenotypic relationship between these MRI metrics and height in our sample. To this end we correlated the global MRI metrics of SA, NDI and CT with standing height measured in cm. In line with previous studies, we found a strong positive correlation between height and SA, and no significant correlation between height and CT or NDI [18, 19, 20, 21].

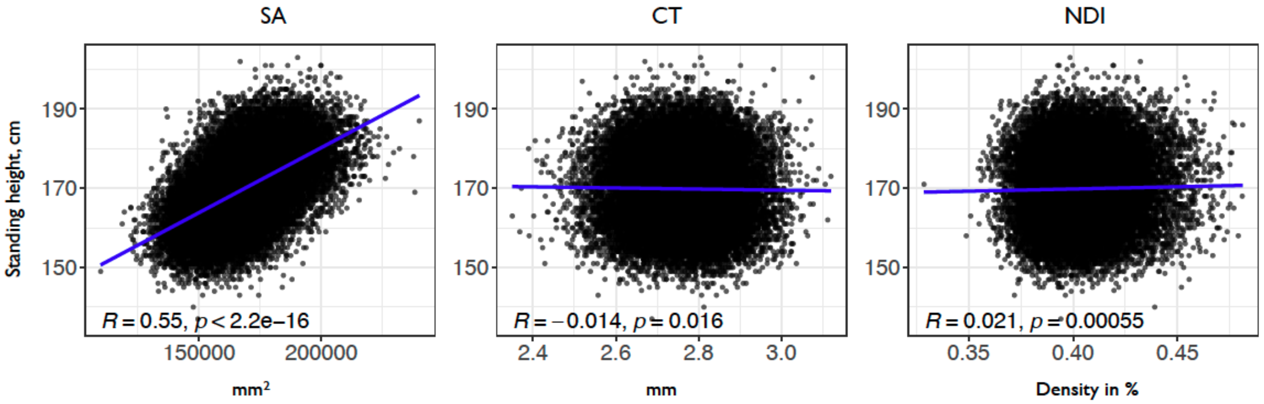

Figure S10. Phenotypic relationships between height and MRI metrics. Shown are two-tailed Spearman's correlations between standing height (y-axis) and global surface area (SA, mm^2), cortical thickness (CT, *mm*) and neurite density index (NDI, density in %). Each point represents one of 31,780 subjects included in the main analyses.

7 Mendelian randomization

As summarised in the main paper, we used Mendelian randomization (MR) analysis to investigate the causal relationships between genetically coupled phenotypes, brain structure and schizophrenia, each of which could be regarded as both outcome and exposure. For the principal analysis, we used invariance weighted (IVW) estimators of MR model parameters. There was no evidence for schizophrenia exposure causing brain change outcomes; however, there was some evidence for genetically determined brain changes (exposure) causing schizophrenia (outcome). Specifically, there were significant causal effects based on the invariance-weight method in two brain regional phenotypes: ProS surface area and V4 surface area.

Invariance-weight (IVW) estimators of MR models assume that all SNPs are valid genetic instruments and that there is no horizontal pleiotropy and is thus not robust to horizontal pleiotropy [22]. Horizontal pleiotropy refers to the fact that a genetic variant (instrument) can be independently associated with multiple phenotypes. For example, a genetic variant can be associated with the outcome by a causal pathway through the exposure, and through an alternative causal pathway that does not include the exposure. Such horizontal pleiotropy contravenes one of the basic assumptions of MR analysis and can bias its results [23]. To assess the robustness of significant findings obtained using IVW and to investigate horizontal pleiotropy we conducted a series of sensitivity analysis.

We repeated our analysis using two robust MR methods that relax the assumption that there is no horizontal pleiotropy: the weighted median method (WM) and MR-Egger. WM provides consistent results even when 50% of the genetic instruments are invalid [22]. MR-Egger is a pleiotropy-robust method that allows for (some) directional pleiotropy, by including an intercept term in the IVW model. The slope from an MR-Egger regression represents the MR-Egger estimate of the causal effect. The intercept in an MR-Egger regression model is zero in the ideal case of no horizontal pleiotropy and significantly non-zero MR-Egger intercepts indicate substantial horizontal pleiotropy [22, 23, 24]. We additionally assessed heterogeneity of the genetic instruments using Cochran's Q value. If there is no horizontal pleiotropy, the MR estimates of causality for each individual SNP should be consistent and will only vary by chance. Thus, larger between-instrument heterogeneity, where effect estimates are more different that expected by chance, would indicate violation of the basic assumptions of MR analysis [25].

Two additional sensitivity analyses used included the MR Presso global test, which detects the presence of horizontal pleiotropy [26], and Steiger filtering, which tests for the direction of effect [27]. We also generated four types of plots for visual inspection: (i) scatter plots showing the SNP effects on exposure versus SNP effects on the outcome. (ii) forest plots showing the variant-specific causal estimate for each individual genetic instrument (also known as Wald ratios) [28], combined with the overall estimates. (iii) leave-one-out plots showing the estimated causal effect of the exposure on the outcome after the exclusion of each genetic instrument, combined with the overall IVW; and (iv) funnel plots displaying the individual Wald ratio for each SNP versus its precision. Plots (i-iii) were used to detect genetic instruments that were potential outliers. Plot (iv) was used to assess unbalanced horizontal pleiotropy, which could bias the results of MR analysis [23, 29], and would be indicated by an asymmetric distribution of the variants around the estimate.

For ProS (prostriate cortex, an area of posterior cingulate cortex, the MR-Egger intercept (P =0.16), the Q-test (P =0.52) and the global MR Presso (P =0.49) tests were not significant, suggesting no evidence for horizontal pleiotropy; and the Steiger test indicated correct causal direction ($P \leq 0.0001$). In addition, leave-one-out analyses did not indicate that the results were driven by any one genetic variant (Fig. S11). The results from ProS SA MR modeling are technically robust and indicate one cortical location of brain-mediated genetic risk for schizophrenia.

For V4, an area of ventral occipital cortex specialised for colour vision, the sensitivity analyses were less consistent. The Egger intercept P =0.09 did not reach significance but approached significance, implying that there might be pleiotropy present. Additionally, the Q-test was significant P =0.01, thus indicating horizontal pleiotropy. However the global MR Presso test was not significant P =0.1 and the Steiger test indicated correct direction of effect $P \leq 0.0001$). The results from V4 SA MR modeling should be regarded with caution in terms of their causal significance.

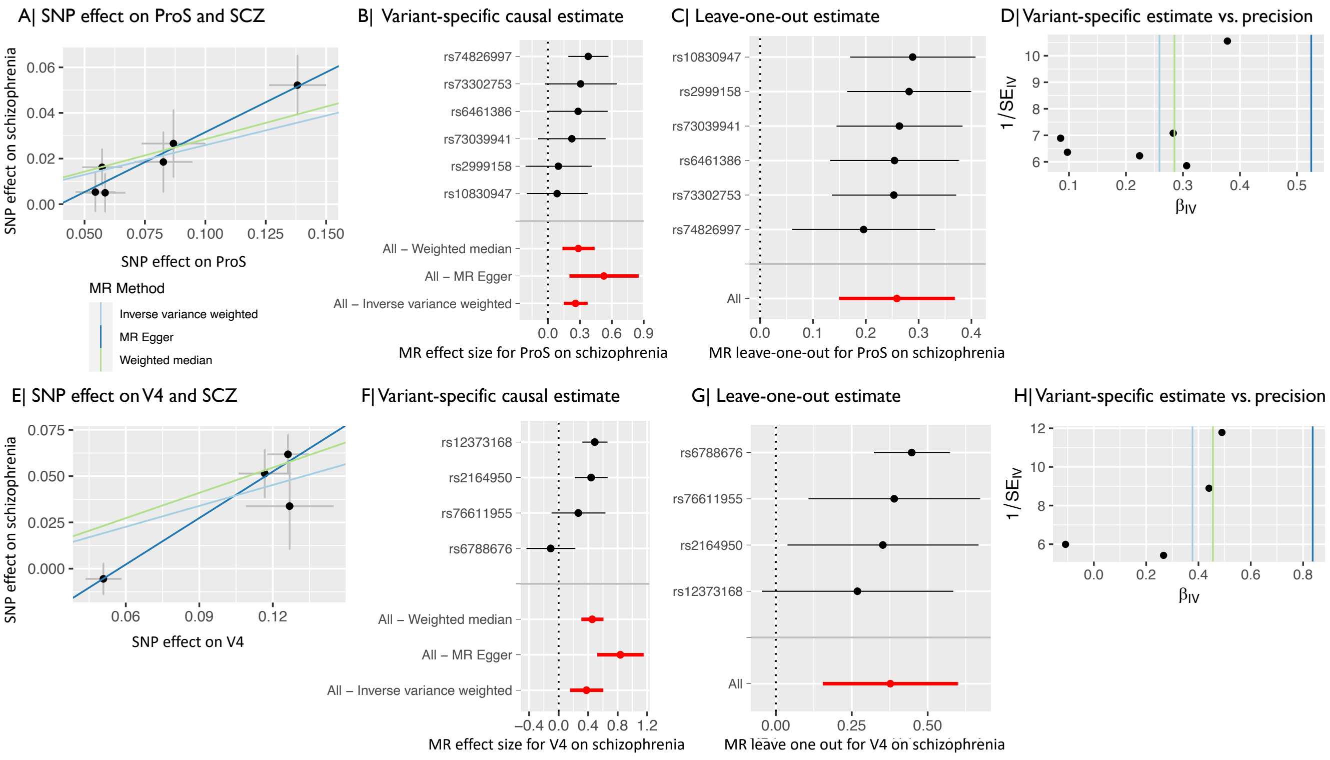

Figure S11. Mendelian randomization plots for surface area of cortical areas, ProS and V4. Results for prostriate surface area (ProS SA) are shown in the top row, results for V4 SA in the bottom row. (A,E) Scatter plots showing the SNP effect on exposure (x-axis) and on the outcome (y-axis). The regression lines represent the causal estimates based on IVW (light blue), MR Egger (blue) and the weighted median method (green). (B,F) Forest plots showing the Wald ratios (i.e. variant-specific causal estimate, x-axis) of each individual genetic instrument, combined with the overall causal estimates for all three methods. (C,G) Leave-one-out plots showing the causal estimate (x-axis) after the exclusion of each genetic instrument, combined with the overall IVW estimate. (D,H) Funnel plots displaying the individual Wald ratio for each SNP (x-axis) against their precision (y-axis).

References

1. Sudlow, C. *et al.* Uk biobank: an open access resource for identifying the causes of a wide range of complex diseases of middle and old age. *PLoS medicine* **12**, e1001779 (2015).

2. Alfaro-Almagro, F. *et al.* Image processing and quality control for the first 10,000 brain imaging datasets from uk biobank. *Neuroimage* **166**, 400–424 (2018).

3. Warrier, V. *et al.* Genetic insights into human cortical organization and development through genome-wide analyses of 2,347 neuroimaging phenotypes. *Nat. Genet.* (2023).

4. Fischl, B. *et al.* Automatically parcellating the human cerebral cortex. *Cereb. cortex* **14**, 11–22 (2004).

5. Glasser, M. F. *et al.* A multi-modal parcellation of human cerebral cortex. *Nature* **536**, 171–178 (2016).

6. Daducci, A. *et al.* Accelerated microstructure imaging via convex optimization (amico) from diffusion mri data. *NeuroImage* **105**, 32–44 (2015).

7. Hedges, E. P. *et al.* Reliability of structural mri measurements: The effects of scan session, head tilt, inter-scan interval, acquisition sequence, freesurfer version and processing stream. *Neuroimage* **246**, 118751 (2022).

8. Haddad, E. *et al.* Multisite test–retest reliability and compatibility of brain metrics derived from freesurfer versions 7.1, 6.0, and 5.3. *Hum. Brain Mapp.* **44**, 1515–1532 (2023).

9. Knussmann, G. N. *et al.* Test-retest reliability of freesurfer-derived volume, area and cortical thickness from mprage and mp2rage brain mri images. *Neuroimage: Reports* **2**, 100086 (2022).

10. Duff, E. *et al.* Reliability of multi-site uk biobank mri brain phenotypes for the assessment of neuropsychiatric complications of sars-cov-2 infection: The covid-cns travelling heads study. *Plos one* **17**, e0273704 (2022).

11. Trubetskoy, V. *et al.* Mapping genomic loci implicates genes and synaptic biology in schizophrenia. *Nature* **604**, 502–508 (2022).

12. Mesulam, M.-M. From sensation to cognition. *Brain* **121**, 1013–1052 (1998).

13. Schomer, D. L. *et al.* Temporolimbic epilepsy. *Princ. behavioral cognitive neurology* 373 (2000).

14. Yeo, B. T. *et al.* The organization of the human cerebral cortex estimated by intrinsic functional connectivity. *J. neurophysiology* (2011).

15. Mullins, N. *et al.* Genome-wide association study of more than 40,000 bipolar disorder cases provides new insights into the underlying biology. *Nat. Genet.* **53**, 817–829 (2021).

16. Wightman, D. P. *et al.* A genome-wide association study with 1,126,563 individuals identifies new risk loci for alzheimer's disease. *Nat. genetics* **53**, 1276–1282 (2021).

17. Yengo, L. *et al.* A saturated map of common genetic variants associated with human height. *Nature* **610**, 704–712 (2022).

18. Vuoksimaa, E. *et al.* Brain structure mediates the association between height and cognitive ability. *Brain Struct. Funct.* **223**, 3487–3494 (2018).

19. Hofer, E. *et al.* Genetic correlations and genome-wide associations of cortical structure in general population samples of 22,824 adults. *Nat. communications* **11**, 4796 (2020).

20. Grasby, K. L. *et al.* The genetic architecture of the human cerebral cortex. *Science* **367** (2020).

21. Tilot, A. K. *et al.* The evolutionary history of common genetic variants influencing human cortical surface area. *Cereb. Cortex* **31**, 1873–1887 (2021).

22. Bowden, J., Davey Smith, G., Haycock, P. C. & Burgess, S. Consistent estimation in mendelian randomization with some invalid instruments using a weighted median estimator. *Genet. epidemiology* **40**, 304–314 (2016).

23. Sanderson, E. *et al.* Mendelian randomization. *Nat. Rev. Methods Primers* **2**, 6 (2022).

24. Burgess, S. & Thompson, S. G. Interpreting findings from mendelian randomization using the mr-egger method. *Eur. journal epidemiology* **32**, 377–389 (2017).

25. Greco M, F. D., Minelli, C., Sheehan, N. A. & Thompson, J. R. Detecting pleiotropy in mendelian randomisation studies with summary data and a continuous outcome. *Stat. medicine* **34**, 2926–2940 (2015).

26. Verbanck, M., Chen, C.-Y., Neale, B. & Do, R. Detection of widespread horizontal pleiotropy in causal relationships inferred from mendelian randomization between complex traits and diseases. *Nat. genetics* **50**, 693–698 (2018).

27. Hemani, G., Tilling, K. & Davey Smith, G. Orienting the causal relationship between imprecisely measured traits using gwas summary data. *PLoS genetics* **13**, e1007081 (2017).

28. Burgess, S., Dudbridge, F. & Thompson, S. G. Combining information on multiple instrumental variables in mendelian randomization: comparison of allele score and summarized data methods. *Stat. medicine* **35**, 1880–1906 (2016).

29. Galan, D. *et al.* Applying mendelian randomization to appraise causality in relationships between smoking, depression and inflammation. *Sci. reports* **12**, 15041 (2022).
