# Supplementary material for: The genetic relationships between brain structure and schizophrenia
Source: Nat Commun. 2023 Nov 28;14:7820. doi: 10.1038/s41467-023-43567-7 (PMC10684873; doi:10.1038/s41467-023-43567-7)
Supplement: Supplementary file 4 — Reporting Summary [file 41467_2023_43567_MOESM4_ESM.pdf]

nature portfolio

Corresponding author(s): Eva-Maria Stauffer

Last updated by author(s): 31.10.2023

Reporting Summary

Nature Portfolio wishes to improve the reproducibility of the work that we publish. This form provides structure for consistency and transparency in reporting. For further information on Nature Portfolio policies, see our Editorial Policies and the Editorial Policy Checklist.

Statistics

For all statistical analyses, confirm that the following items are present in the figure legend, table legend, main text, or Methods section.

n/a	Confirmed	
☐	☒	The exact sample size (*n*) for each experimental group/condition, given as a discrete number and unit of measurement
☐	☒	A statement on whether measurements were taken from distinct samples or whether the same sample was measured repeatedly
☐	☒	The statistical test(s) used AND whether they are one- or two-sided<br>*Only common tests should be described solely by name; describe more complex techniques in the Methods section.*
☐	☒	A description of all covariates tested
☐	☒	A description of any assumptions or corrections, such as tests of normality and adjustment for multiple comparisons
☐	☒	A full description of the statistical parameters including central tendency (e.g. means) or other basic estimates (e.g. regression coefficient) AND variation (e.g. standard deviation) or associated estimates of uncertainty (e.g. confidence intervals)
☐	☒	For null hypothesis testing, the test statistic (e.g. *F*, *t*, *r*) with confidence intervals, effect sizes, degrees of freedom and *P* value noted<br>*Give P values as exact values whenever suitable.*
☐	☒	For Bayesian analysis, information on the choice of priors and Markov chain Monte Carlo settings
☐	☒	For hierarchical and complex designs, identification of the appropriate level for tests and full reporting of outcomes
☐	☒	Estimates of effect sizes (e.g. Cohen's *d*, Pearson's *r*), indicating how they were calculated

Our web collection on statistics for biologists contains articles on many of the points above.

Software and code

Policy information about availability of computer code

Data collection	MRI data for this project came from the UK Biobank. GWAS summary data were generated by other researchers. This project was based on secondary data.
Data analysis	Statistical analysis was performed using R version 3.5.2. Code is available on Github: https://github.com/evastauffer/schizophrenia-and-brain-structure; Software used included: Plink version 1.9 https://zzz.bwh.harvard.edu/plink/plink2.shtml ; twosampleMR' package v0.5.6 https://mrcieu.github.io/TwoSampleMR/articles/introduction.html ; LAVA https://github.com/josefin-werme/LAVA ; FUMA version 1.6.0 https://fuma.ctglab.nl/ ; H-MAGMA https://github.com/thewonlab/H-MAGMA ; gProfiler2 https://cran.r-project.org/web/packages/gprofiler2/index.html

For manuscripts utilizing custom algorithms or software that are central to the research but not yet described in published literature, software must be made available to editors and reviewers. We strongly encourage code deposition in a community repository (e.g. GitHub). See the Nature Portfolio guidelines for submitting code & software for further information.

Data

Policy information about availability of data

All manuscripts must include a data availability statement. This statement should provide the following information, where applicable:

- Accession codes, unique identifiers, or web links for publicly available datasets
- A description of any restrictions on data availability
- For clinical datasets or third party data, please ensure that the statement adheres to our policy

Data used in this project are available as outlined below. Summary statistics of cortical MRI phenotypes are available for download from https://portal.ide-cam.org.uk/overview/483. Summary statistics for schizophrenia, bipolar disorder and Alzheimer's disease can be accessed from the Psychiatric Genomics Consortium https://pgc.unc.edu/for-researchers/download-results/. Height GWAS results are available at https://www.nature.com/articles/s41586-022-05275-y. MRI Data from the UKB can be applied for and accessed by approved researchers https://www.ukbiobank.ac.uk/. Spatiotemporal gene expression data can be accessed from PsychENCODE under http://development.psychencode.org/files/processed_data/RNA-seq/mRNA-seq_hg38.gencode21.wholeGene.geneComposite.STAR.nochrM.gene.count.txt. Cell type specific expression data can be downloaded from http://solo.bmap.ucla.edu/shiny/webapp/. Information on constrained genes can be accessed from gnomAD https://gnomad.broadinstitute.org/downloads#v2-constraint. Gene sets for positional enrichments (MSigDB C1, version MSigDB 2023.2.Hs) can be found at https://www.gsea-msigdb.org/gsea/msigdb/human/collection_details.jsp#C1. Source data are provided with this paper.

Research involving human participants, their data, or biological material

Policy information about studies with human participants or human data. See also policy information about sex, gender (identity/presentation), and sexual orientation and race, ethnicity and racism.

Reporting on sex and gender	We use the term sex throughout, and in our study this refers to genetic sex, determined by the composition of sec chromosomes.
Reporting on race, ethnicity, or other socially relevant groupings	We restricted our analyses to individuals of predominantely European ancestry as inferred by genetic principal components. This is also referrd to as genetically referred ancestry.
Population characteristics	The MRI sample were based on subjects of the UK Biobank. The UK Biobank is a population-based prospective cohort of 500,000 individuals from the UK, aged between 39 and 73 years. Approximately 40,000 scans have been completed when the current study commenced.
Recruitment	Participants from the UK Biobank were recruited by other researchers. Details of how participants were recruited are provided in papers references in the Methods section.
Ethics oversight	Ethical procedures for the UK Biobank are controlled by the Ethics and Guidance council (http://www.ukbiobank.ac.uk/ethics), and the study was conducted in accordance with the UK Biobank Ethics and Governance Framework document (https://www.ukbiobank.ac.uk/media/0xsbmfmw/egf.pdf), with institutional review board approval by the North West Multi-center Research Ethics Committee.

Note that full information on the approval of the study protocol must also be provided in the manuscript.

Field-specific reporting

Please select the one below that is the best fit for your research. If you are not sure, read the appropriate sections before making your selection.

☒ Life sciences ☐ Behavioural & social sciences ☐ Ecological, evolutionary & environmental sciences

For a reference copy of the document with all sections, see nature.com/documents/nr-reporting-summary-flat.pdf

Life sciences study design

All studies must disclose on these points even when the disclosure is negative.

Sample size	We focused on a subset of N = 40,680 participants for each of whom complete genotype and multimodal MRI data were available for download (February 2020). We excluded participants with incomplete MRI data and we additionally excluded participants who were robustly defined as outliers by global or regional metrics more than 5 times the median absolute deviation from the sample median (± 5 MAD). For CT and SA this lead to 31,780 subjects and 31,797 subjects for NDI.
Data exclusions	We excluded participants with incomplete MRI data and we additionally excluded participants who were robustly defined as outliers by global or regional metrics more than 5 times the median absolute deviation from the sample median (± 5 MAD). For CT and SA this lead to 31,780 subjects and 31,797 subjects for NDI.
Replication	We replicated the genetic intersection between schizophrenia and both surface area and cortical thickness on chromosome 17q21.31 using previously published lists of high confidence genes by other researchers.

Randomization	No randomisation as all data were from observational studies
Blinding	Blinding is not relevant as this is an association study

Reporting for specific materials, systems and methods

We require information from authors about some types of materials, experimental systems and methods used in many studies. Here, indicate whether each material, system or method listed is relevant to your study. If you are not sure if a list item applies to your research, read the appropriate section before selecting a response.

Materials & experimental systems

n/a	Involved in the study
☒	☐ Antibodies
☒	☐ Eukaryotic cell lines
☒	☐ Palaeontology and archaeology
☒	☐ Animals and other organisms
☒	☐ Clinical data
☒	☐ Dual use research of concern
☒	☐ Plants

Methods

n/a	Involved in the study
☒	☐ ChIP-seq
☒	☐ Flow cytometry
☒	☐ MRI-based neuroimaging

Plants

Seed stocks	*Report on the source of all seed stocks or other plant material used. If applicable, state the seed stock centre and catalogue number. If plant specimens were collected from the field, describe the collection location, date and sampling procedures.*
Novel plant genotypes	*Describe the methods by which all novel plant genotypes were produced. This includes those generated by transgenic approaches, gene editing, chemical/radiation-based mutagenesis and hybridization. For transgenic lines, describe the transformation method, the number of independent lines analyzed and the generation upon which experiments were performed. For gene-edited lines, describe the editor used, the endogenous sequence targeted for editing, the targeting guide RNA sequence (if applicable) and how the editor was applied.*
Authentication	*Describe any authentication procedures for each seed stock used or novel genotype generated. Describe any experiments used to assess the effect of a mutation and, where applicable, how potential secondary effects (e.g. second site T-DNA insertions, mosiacism, off-target gene editing) were examined.*
